# Stay the course: practitioner reflections on implementing family-based treatment with adolescents with atypical anorexia

**DOI:** 10.1186/s40337-019-0240-8

**Published:** 2019-04-25

**Authors:** Gina Dimitropoulos, Melissa Kimber, Manya Singh, Emily P. Williams, Katharine L. Loeb, Elizabeth K. Hughes, Andrea Garber, April Elliott, Ellie Vyver, Daniel Le Grange

**Affiliations:** 1 Faculty of Social Work, University of Calgary, 2500 University Drive NW, Calgary, Alberta T2N 1N4 Canada; 2 Department of Psychiatry and Behavioural Neurosciences, McMaster University, Hamilton, Canada; 3 Werklund School of Education, University of Calgary, Calgary, Canada; 4 School of Psychology, Farleigh Dickinson University, Teaneck, New Jersey USA; 5 Department of Paediatrics, University of Melbourne, Melbourne, Australia; 6 Murdoch Children’s Research Institute, Melbourne, Australia; 7 Centre for Adolescent Health, Royal Children’s Hospital, Melbourne, Australia; 8 Department of Pediatrics, University of California, San Francisco, California USA; 9 Department of Pediatrics, University of Calgary, Calgary, Canada; 10 Departments of Psychiatry and Pediatrics, University of California, San Francisco, California USA; 11 Department of Psychiatry and Behavioral Neuroscience (Emeritus), The University of Chicago, Chicago, Illinois USA

**Keywords:** Eating disorders, Atypical anorexia nervosa, Family-based treatment, Weight restoration, Treatment outcomes

## Abstract

**Background:**

Atypical anorexia nervosa (AN) has received minimal empirical attention regarding effective diagnosis and treatment. Family-based treatment (FBT) might be a promising treatment for atypical AN, yet it is unclear as to what adaptations are needed to the current manualized FBT for AN model. The objective of the current study was to identify how FBT practitioners applied FBT for atypical AN for adolescents in their clinical practice, and if there were any implementation challenges and adaptations to the model for this population.

**Methods:**

The current study employed fundamental qualitative description, with the aim of capturing practitioners’ reflections on working with adolescents with atypical AN in clinical practice. A purposeful sample of practitioners with training in FBT were recruited and each participant completed an individual, semi-structured interview. Data was analyzed using conventional content analysis.

**Results:**

A total of 23 practitioners participated in this study. The results indicate that practitioners maintained some fidelity to manualized FBT in treating atypical AN, but they differed in their discussions around target weights, what constitutes weight restoration, and the dosage for FBT phases. Salient practice challenges included operationalizing the *Diagnostic and Statistical Manual of Mental Disorders – 5th Edition* (DSM-5) definition of atypical AN, identifying a ‘goal weight’ for adolescents and activating parents to take charge of the re-nourishment process.

**Conclusions:**

The results of this qualitative study demonstrate practitioner reflections on the delivery and adaptations of FBT for adolescents with atypical AN. These reflections highlight the need to establish the delivery of coherent and consistent treatment and messaging with patients and families. Further, practitioners’ reflections highlight common strategies to increase the sense of urgency in parents to support their child with atypical AN.

## Plain English summary

This manuscript describes the qualitative data analysis of 23 interviews with practitioners who use Family-Based Treatment (FBT) when working with adolescents with atypical anorexia nervosa (AN). Due to the recency of the classification of atypical AN in the DSM-5, little is known regarding effective treatments for this population. Given the literature supporting FBT as an effective form of treatment for adolescents with AN, the objective of the current study was to identify how FBT practitioners applied FBT for atypical AN for adolescents in their clinical practice, and whether any adaptations were made to the FBT model for this population. Thus, this study provides important insights on practitioner reflections on the application of FBT with adolescents with atypical AN. The application of existing treatments is important to understand so that practitioners have an approach to use with adolescents who present with atypical AN in treatment.

## Background

Atypical anorexia nervosa (AN) is a serious eating disorder that is commonly found in community and clinical samples [1, 2]. Atypical AN occurs when an individual meets all the criteria for AN, but despite dramatic and significant weight loss, their weight is within or above the normal range ([3], p. 353). Prior to the *Diagnostic and Statistical Manual for Mental Disorders – 5*^*th*^ *Edition* (DSM-5), individuals with such symptomatology were often grouped in the Eating Disorder Not Otherwise Specified (EDNOS) category or referred to as exhibiting ‘sub-threshold’ AN. However, since the publication of the DSM-5, individuals with these criteria have been classified under atypical AN, making it difficult to disentangle whether literature prior to the DSM-5 included cases true to atypical AN or other eating disturbances more broadly. In this manuscript, we will refer to cases which: (a) meet DSM-5 diagnostic criteria for atypical AN, and; (b) manifest eating disturbances that were once classified as EDNOS but have been re-conceptualized as atypical AN based on current understanding.

Studies have found comparable genetic risk factors for typical and atypical AN [4]. Other research shows that adolescents with atypical AN have higher rates of anxiety and suicidality [1], and ED-related cognitions and obsessive-compulsive behaviors [5] than those with typical AN. With regard to physical complications, a recent study by Sawyer et al. [6] compared adolescents with typical and atypical AN and found no differences in medical instability. In addition, individuals with typical and atypical AN present with similar cardiovascular complications and laboratory results when hospitalized [6, 7]. Those with atypical AN tend to report significant eating disorder cognitions, body dissatisfaction and distress about shape/weight [6] and psychosocial impairment [8–13]. To date, there are minimal studies evaluating interventions for adolescent populations with atypical AN [2, 14–16], as such, treatment recommendations specific to atypical AN have yet to be developed which would allow practitioners working with this population to effectively adapt within current evidence based practice.

According to the UK National Institute for Health and Care Excellence, Family-Based Treatment (FBT) is considered the most effective intervention for adolescents with AN [17]. Researchers have also acknowledged that FBT may be a helpful model to apply to atypical AN populations [18]. FBT for AN seeks to create a sense of urgency for parental involvement to address malnutrition, ensuring full weight restoration though normalizing eating and extinguishing compensatory behaviours. Manualized FBT consists of three distinct phases; in phase 1, parents are charged with the responsibility to re-nourish their child back to health by supporting them during meals. In phase 2, parents incrementally relinquish the control over meals and food choices, as these are returned back to the child. In the final phase of FBT, the young person and their family review their progress in treatment and address any outstanding adolescent developmental issues.

That said, adolescents with atypical AN may not be underweight with respect to their BMI or a similar measure, but they may be underweight in terms of what is healthy for them based on their personal growth history [6]. Apart from differences in weight and the need for weight gain, adolescents with atypical AN have other symptoms to address in phase 1 of treatment, such as normalization of food and compensatory behaviours; this renders FBT manuals for typical AN potentially less appropriate for the atypical AN patient population. It is possible that adaptations to FBT for AN would be required to propel parents into treatment when AN behaviors may be transient and the degree of weight loss is perceived to less physically threatening.

Recently, Hughes and colleagues [19] conducted a case series of 42 adolescents (age 12–18, 88% female) with atypical AN treated with FBT for AN. Weight gain and maintenance is typically considered a requirement for recovery in FBT for AN, however at the end of treatment, one adolescent remained below population-based expected weight for their height. In addition to using weight, ED psychopathology was considered when defining remission post-treatment in this study. Overall, engagement in the study trial was high, with 83% of the sample completing at least half of the treatment dose. Of those completing the treatment, remission ranged from 38 to 52% depending on whether cognitive symptoms and/or weight criterion were considered. This is promising evidence that FBT may be an effective model for the treatment of atypical AN. Hughes and colleagues’ [19] trial suggested that a broader perspective of atypical AN be considered, namely that weight status should only be one factor, and potentially not the deciding factor, for this patient group.

### Study purpose

With an increasing number of adolescents presenting with atypical AN in clinical samples [20], there is an urgent need for effective interventions for this population. Although FBT is an acceptable and effective model of treatment for adolescents with AN, it remains unclear as to if this treatment modality can meet the needs of adolescents with atypical AN, and if so, what adaptations (if any) are necessary. The primary objectives of the present study were to: (a) elucidate how FBT practitioners conceptually distinguish typical and atypical AN; (b) explore FBT practitioners’ perceptions and experiences of implementing FBT for adolescents with atypical AN; (c) determine what, if any, adaptations to the FBT model practitioners make for atypical AN adolescents, and; (d) uncover any unique practice challenges practitioners face while implementing the FBT model with this patient population. Practitioner reflections on the delivery of FBT for the treatment of adolescents with atypical AN were sought.

## Methods

This qualitative, exploratory study was informed by the methodological principles of fundamental qualitative description. Fundamental description allows for the detailed and systematic capturing of experiential information in applied health care contexts to inform practice-based changes [21]. This method also allows for innovative approaches to intervention to meet the needs of a specified patient population [22]. Fundamental qualitative description is utilized to be descriptive rather interpretative in nature. We elected to use fundamental qualitative description to help us explore current practices when applying FBT for atypical AN. Therefore, we were interested in how, rather than why, they practice in a certain way with this patient population. This study was approved by the University of Calgary Institutional Ethics Board.

### Setting and sample

The current study was conducted at the University of Calgary in Calgary, in Alberta, Canada. A purposeful sample of practitioners were recruited using multiple methods. First, a roster of practitioners who had undergone introductory training in FBT through the Training Institute for Child and Adolescent Eating Disorders, Limited Liability Company (LLC; hereafter referred to as the “Training Institute”) were invited to participate via email invitation. Second, the research team was encouraged to forward and share the respective recruitment materials with practitioners at their organizations. The recruitment email and materials indicated that anyone interested in participating should get in touch with the principal investigator^1^ (PI) of the study. Third, upon completion of a participant’s interview, these individuals were asked to share the recruitment materials with individuals they thought may be eligible and interested in participating in the study – a purposeful sampling strategy termed snowball sampling [23].

We estimated that a sample of ~ 20 practitioners would yield a robust pattern of perceptual and experiential information in the data. Our sample size was based on previous research suggesting that qualitative studies which utilize inductive methodologies (e.g., grounded theory methodology) include about 20 to 30 participants in their samples (see Creswell [24]), and that new information is seldom derived from qualitative interviews after 20 or more participants [25].

A total of 23 practitioners participated in this study. Given that the goal of this study was to provide an initial description of practitioner experiences of implementing FBT with adolescents with atypical AN, information pertaining to practitioner setting was not systematically collected. Rather, we collected information pertaining to the practitioner’s discipline. Among the participants who provided information about their practice setting, five indicated that they were in private practice, and one indicated that they were working in a psychiatric hospital. Approximately 50% of the participants who disclosed their age (*n* = 21; missing data: *n* = 2) reported being less than 40 years, with an overall age range from 32 to 52 years. Twenty participants reported the number of years they had been implementing FBT in their practice, with a range of two to 16 years. The total number of years practicing in eating disorders (EDs) ranged from three to 18 years (*n* = 19). Practitioners noted that the proportion of adolescents with atypical AN enrolled in FBT in their practice ranged from three to 50%.

### Data collection

Only practitioners who had experience working with adolescents within the context of FBT were included in this study. Participants were invited to complete a semi-structured interview with the PI of the study (first author). Interviews were conducted over the phone and transcribed verbatim by the research team and lasted between 45 and 90 minutes. Interview guides were used to focus and explore specific areas, including: (a) practitioners’ perceptions and experiences of working with and delivering FBT to adolescents with typical versus atypical AN; (b) what adaptations (if any) practitioners made to the FBT model when working with adolescents with typical versus atypical AN, and; (c) factors or characteristics that practitioners perceived as important when working with adolescents with typical versus and atypical AN and their families.

### Data analysis and methodological rigor

Interview transcripts were analyzed using conventional content analysis. Conventional content analysis involved the processes detailed by Elo and Kyngas [26]. Specifically, the second author reviewing each transcript in its entirety, then completing line-by-line hand-coding of the textual data within and across transcripts to capture key concepts (called: *codes*) that were discussed in the interviews. The second author then collapsed the codes into broader, overarching categories, compiled a codebook detailing each category, its definition and its characteristics for the classification of the data and submitted for review and approval by the PI to ensure the dependability of the data analysis. Approval of the codebook required that the PI independently review a portion of the study transcripts to discern whether or not the developed categories, definitions and characteristics were appropriate given the data provided by the research participants and the objectives of the research study. The PI approved all categories included in the codebook. The codebook was then applied to all transcripts in QSR Nvivo 10 by the second author’s research assistant, a process that was reviewed and verified by the second author. In addition, interim member-checking of the data was performed by the PI of the study, such that questions or ideas present in previous interviews were posed to FBT therapists who did not participate in the study to determine if the preliminary findings resonated for them.

Our choice of analytical strategy is supported by qualitative methodological work in the health sciences. For example, a seminal paper by Hsieh and Shannon [27] in the journal of *Qualitative Health Research* details the three most commonly used forms of content analysis to make meaning from qualitative data, which include conventional, directed, and summative content analysis. Conventional content analysis [27] is an inductive approach to qualitative data analysis that does not assume a pre-existing explanation for the experience of participants in a given study. Rather, analysis allows for categories to inductively emerge from within and across the collected data and focuses on the interpretive possibilities of the findings – an approach which is followed in our study and is also supported by methodological papers detailing Fundamental Qualitative Description.

## Results

Conventional content analysis revealed the following categories from the interviews with the practitioners: operationalizing atypical AN; tenets of FBT, and; challenges specific to the processes in FBT. Within these categories, sub-categories were developed to demonstrate practitioners’ perceptions about the conceptualization of atypical AN, the utility of FBT, the dose of the treatment for atypical AN, and the challenges of employing FBT with adolescents with atypical AN and their families. Refer to Table 1 for illustrative quotes supporting code development.

**Table 1:** Illustrative Quotes Supporting Code Development and Reporting

Code	Sample Quotes
Operationalizing Atypical AN	
Variability in Weight Criteria	“…It’s someone who loses weight in an eating disordered way and the final weight they get to is not underweight by whatever standards we usually measure that. It might be very unhealthy for that person, but maybe they’re 50th percentile BMI or higher and they started out at the 90th percentile BMI.” (Par 10, pg. 2)
	“So, I guess when I think about it, I’m thinking about primarily weight. So, the patient may not be significantly underweight compared to other kids their age, yet they’re underweight for their bodies and they’ve lost a significant amount of weight and they’re displaying the features of AN that we typically see in more underweight patients” (Par 11, pg. 1)
	“One is the kids that, who according to their BMIs, their doctors were categorizing them as overweight, or very overweight or whatever it is and then they lose a massive amount of weight especially in a short period of time, but at the time they weren’t meeting the criteria for anorexia. So, there is sort of that category and then there’s the other kids that were caught very quickly and they just haven’t lost that much weight yet and yet they are certainly demonstrating all of the symptoms except for that extreme weight loss. Those are the two clusters that I have in mind” (Par 09, pg. 1)
Weight is One Piece of the Medical Puzzle	“Sometimes we look at the rigidity of their thinking as well, because I find if it’s more of an atypical kind of pattern, then they are not super starved which is what you see in the anorexia and then the rigidity and thinking isn’t as strong [as typical AN]” (Par 08, pg. 2).
	“I think a lot of times they are just expressing concern that the person has been not really eating as much as they used to be eating. They have noticed a lot of rigidity around food, so going to the quote on quote health food versus eating a variety of food. They have seen more irritability, signs of depression and anxiety and those types of things that they are having trouble making sense of. I think most of the time they say this is someone we have never had any problems with, she has always been really high functioning, she has always done really well for herself, and then she seems to like, her personality has really shifted.” (Par 06, pg. 2)
Tenets of FBT	
Presentation of Exercise (compared to typical AN adolescents)	“Interviewer: Are they going back to exercising much more quickly?
	Participant: Not necessarily. You know, when I work with these cases, the paediatrician and I guess, we decide together when a child can exercise, but it really is once they are doing much better, you know, the weight is up, there is not much resistance with eating.” (Par 19, pg. 4)
	“You know, I think it’s about the same, they are…I’m just trying to think…I would say they’re about the same, I am just trying to think of the last few that we assessed and I would say that in terms of exercise being an issue, it is about the same” (Par 08, 09)
Reintroduction of Exercise (compared to typical AN adolescents)	“Yeah, I feel like I am more lenient in my level of like my recommendations about activity. First of all, I think the family pushes it a lot – let’s find health ways to control weight – and I find that I am probably a little more lenient than with some of the other, like it seems like kind of a normal thing to do…It seems a little protective to me. Like why don’t we help them to manage some aspects of weight in a healthy way while they are still in treatment? And, I have less of a concern unless the way they lost their weight was through excessive activity…I find I feel more comfortable with them doing it while they are still in treatment and so it seems kind of more in line with the overall messaging of, ‘yes, we want to kind of promote health, but it is a healthy kind of balance of making sure you are eating enough…’ especially if they have been quite significantly overweight in the past” (Par 23, 8).
	“They can’t keep doing this [exercise] no matter who they are [i.e, typical or atypical]. So the answer is not like, ‘because they have atypical AN, some level of exercise is okay in phase 1.’ No, I would never make that change” (Par 01, pg. 6)
Weighing the Patient and Graphing the Weight Chart	“And I guess, I’m sensitive to the patient’s weight gain and their realistic fears about becoming overweight again. I think I’m more individualised about the way I present growth curves to families, whether I involve the patient in that conversation initially or not; it’s just, I don’t have one particular way of doing it, but I feel like with Atypical AN, it’s okay to sort of like deviate a little bit from the typical way of doing it” (Par 10, pg. 3)
	“I show their weight curve and I talk about how you know ‘you are underweight.’ And that really confuses them a lot when I am like ‘you’re really underweight, you’re malnourished’ and they’re like, ‘I’m not underweight, I’m at the 50th percentile.’ I’m like, ‘for you, you are underweight’ and kind of showing [individual] growth curves. Parents…the curve, being able to show that data, I think helps them because sometimes it is quite dramatic.” (Par 12, pg. 9)
Challenges Specific to Processes in FBT	
Activation of the Parental Dyad and Creating an Intense Scene	“I feel like we have had these parents where we have gotten a set of instructions from the doctors about losing weight and have done everything right and have done everything that they were supposed to do and now they are being told, this isn’t okay you need to refeed them [the child]. They are just totally confused” (Par 18, pg. 1).
	“I mean often times parents will, you know, notice how much the child has changed since the weight loss, and they can identify the eatingdisorder behaviours that are so atypical, or out of character, like new to the family, but I think there is less sort of identification of the eating disorder voyage as really being…you know, like sometimes with very low weight patients, like the parents are like ‘this is nuts! She still thinks she’s fat?’ and like ‘look at her!’. So, its like they can kind of identify the eating disorder as really being all the more pathological, almost like psychotic. Whereas, I think that with a patient with atypical AN, the parents maybe can understand a little bit more why the patient is concerned about their weight, you know if a patient goes from being the biggest kid in the class to maybe a little heavy, to being svelte and looking a little more like her peers, I think the parents, its maybe harder for them to understand that it’s so disordered to want to look like that…I guess yea, the parents have more empathy for the eating disorder” (Par 11, pg. 6).
Allied Health Professionals – Disciplinary Differences	“Physicians are not really sure why the families have been referred for treatment, I feel like they see it as it conflicts with what their recommendation was and I don’t know if at times they feel like they are on the defensive like we are attacking them saying, ‘this should not have happened’ and they are saying ‘it should have happened, they [the adolescent] were overweight and they needed to lose weight.’ So I think a lot of times maybe their perception is that I am wanting to get the teen back to where they were in an overweight or obese place and I have to also give them the message that ‘no, we are not asking to re-nourish and be at an unhealthy weight, we are just wanting to make sure that we find the right balance for this teenager” (Par 22, pg. 1).
	“And you know, as the therapist, you don’t want to disrespect the physician you know and sort of say ‘he doesn’t know what he is talking about’ but at the same time, you know I think sometimes I’ve had to say to parents very respectfully and very kindly that even very very good physicians don’t always get training in eating disorders and this is what we’re saying as opposed to what they’re saying and how can we all get on the same boat here?” (Par 13, pg. 5)
	“They [parents and adolescents] are confused…and often, this is so sad, often these things can start from pediatricians who are well-meaning and they are saying hmmm, we need to watch your weight, maybe go on a diet, and before you know it, we have a really major health risk.” (Par 09, pg. 5).
Determining the Goal Weight for Atypical AN Adolescents	“I’m often relying on as much behavioral evidence as I can, ‘hey this is what they’re [your child] is eating, maybe this [something different] was what they were eating before the eating disorder, and that wasn’t ideal either, however, this is what they’re doing now’ and emphasizing that this is not a phase and then what I think is compounded by all of this stuff is what’s the weight target? So, if we know that a kid needs to gain some weight, but do they need to go all the way back to where they were before? I think this is a time when often we’ll start with the 76th percentile and go up till about the 85th percentile and sort of see where we are at.” (Par 03, pg. 1).
	“I do think there is a little less to guide us with these kids as far as where their weight should be so sometimes I’ll say ok, you don’t want to get her up to the 90th percentile, let’s get her up to the 75th and see how she is and I think, there’s a couple of things. One, I think it gets them [the parents] at least on board of starting the process of weight regain, but it also gives the therapist and the treatment team a little bit of wiggle room because I think it’s less clear where these kids should be.” (Par 13, pg. 13).

### Operationalizing atypical AN

Practitioners explicitly acknowledged that there was a lack of certainty around how atypical AN was operationalized and assessed in practice, particularly in relation to the extent of weight loss the adolescent experienced prior to presenting for care. All practitioners grounded their definition of atypical AN in *relation to* cases that did not meet ‘weight criterion’ for the diagnosis of typical AN (i.e., patients presenting with diagnostic criteria for AN, but despite significant weight loss fell within the normal or above average weight range). Although practitioners were consistent in conceptualizing atypical AN patients as not meeting the diagnostic weight criterion of typical AN in the DSM-5, they were inconsistent in their understanding of what constituted significant weight loss in relation to their patients’ weight trajectories. Regardless of this uncertainty, practitioners were consistent in describing that the adolescent’s weight is “one piece of the medical puzzle” (Par 05, pg 3). In addition, practitioners noted that a number of other indicators were considered in their assessment and classification of atypical AN, including: heart rate; body temperature; blood pressure; changes in skin and hair texture; drive for thinness; body dissatisfaction; extent of restricted eating; the presence, absence and severity of other disordered eating behaviors (intentional vomiting, laxative use, excessive exercise, et cetera), and; mood and anxiety symptoms. Practitioners in this study did not determine a threshold for the above-mentioned indicators or use standardized scales to assess indicators in terms of atypical AN classification. However, practitioners did mention that they assessed for certain eating disorder-related issues (e.g., body dissatisfaction), as they commonly observe these symptoms in those with typical AN.

### Key tenets of FBT

With respect to a possible intervention for adolescents with atypical AN, practitioners indicated that one should ‘stay the course’ where FBT is concerned, and were decisive in their description of FBT as an appropriate first-line intervention for this adolescent population. With a few exceptions (*n* = 2), practitioners in our study indicated that weight-gain was a necessary component for the treatment of atypical AN and a prerequisite for reducing eating-disordered cognitions. Practitioners were also asked to share their perspectives of the extent to which they felt they implemented the tenets of FBT differently or similarly when working with adolescents with atypical AN. With respect to the externalization of the illness, as well as the completion of the family meal, practitioners were explicit that these key tenets were analogous for atypical and typical AN. Practitioners also took a similar position on the importance of restricting exercise during Phase 1 for adolescents with typical and atypical AN, particularly for those who were medically unstable.

#### Weighing the patient and graphing the weight chart

In the foundational model of FBT, the practitioner weighs the patient at the start of every session and charts the weight throughout treatment. In the present study, all practitioners reported routinely weighing adolescents with atypical AN, however, there were inconsistent practices in terms of how practitioners presented weight charts to patients and families. Eight of the 23 practitioners described using the weight chart similarly when treating atypical and typical AN. Yet, 15 practitioners described that they varied this process between these patient populations. Variations for atypical AN included graphing and presenting only individual weight growth trajectories, graphing the adolescent’s weight (e.g., on one line) in relation to population norms for their age and gender (i.e., on another line; note: referring to and graphing population weight norms is not part of FBT practice; [28]), and omitting the presentation of the graph (as well as any discussions of weight) from their clinical encounter altogether. In the latter case, practitioners indicated that their discussion about the adolescents’ weight would strictly focus on the stability of weight (i.e. the extent to which it fell within 1 kg from 1 week to the next). As one FBT practitioner with a social work background stated:

I guess I would say one thing I do differently: I’m not as visual with the growth chart because I think parents and kids will have a lot of sensitivity. So, I’m not sort of pointing out, ‘this is where you were before and this is where we’re going.’ But, I am still focused on weight gain because I think it’s really important and these kids have lost weight…I may not even get it [the growth chart] out. (Par 01, pg. 3)

#### Duration of FBT phases and indicators of completion

Practitioners were equivocal in their descriptions about the extent to which the duration of the phases of FBT treatment varied between typical and atypical AN adolescents. In some cases, practitioners described that Phase 1 of FBT would be shorter if the adolescent with atypical AN lost a small amount of weight and were close to their “normal weight;” there was also the perception that these adolescents gained weight faster. In other cases, however, practitioners noted that Phase 1 could last longer if parents and adolescents struggled to accept the need for intervention. In other words, the first phase of FBT may very in duration depending on the adolescent’s presentation in treatment including, but not limited to, the degree of weight loss. Similarly, practitioners shared that both Phase 2 and 3 moved quicker for some atypical AN adolescents and lasted longer for others.

Despite the equivocal reports around the duration of the FBT phases, practitioners were consistent in their reports of the indicators for phase completion. Markers to begin phase 2 included the decreased emphasis on and rigidity about food, weight restoration, medical symptoms such as stable vital signs or normal bloodwork and parents feeling in control of the feeding and monitoring processes. Practitioners also noted that the family would be near or at completion of phase 2 when it was clear that eating and exercise patterns were normalized for an extended period of time and other issues related to family dynamics (e.g., academics, adolescent individuation, conflicts separate from food, eating or exercise) surfaced for discussion on a regular basis. Phase 3 completion was described as the parent and adolescent giving an indication that they were ready to move on from the therapy process.

### Practice challenges specific to processes in FBT

#### Activation of the parental dyad

A pervasive challenge described across all interviews related to parents’ and adolescents’ inability or willingness to acknowledge the seriousness of the eating disorder, and therefore, recognize the importance of initiation and follow through on treatment. From the participants’ perspectives, this inability was driven by a tendency for adolescents with atypical AN to present at an ‘average’ or ‘normal’ weight and therefore, they did not appear to be sick. As one practitioner stated:

I always find that these cases can be quite difficult to generate the right amount of anxiety to kind of actually push the parents into kind of active mode, so I think that I have to spend a bit more time on that because often they are not seen as emaciated…It is more difficult to generate the right amount of anxiety in the family. (Par 21, pg. 4)

To this end, practitioners indicated that activating the parents to take on the role of nourishing their affected child—as directed by the FBT manual – was perceived as confusing to the adolescents and their families. To counter parents’ and adolescents’ reliance on appearance or weight as the indicator of wellness or illness, practitioners would draw on additional medical markers of atypical AN (e.g., heart rate, body temperature). Relatedly, practitioners indicated that if the adolescent was not demonstrating any medical complications from their weight-loss, this exacerbated the challenge of activating families to legitimize the eating disorder and the immediacy for intervention. In these cases, practitioners felt it was critical to provide parents with information about the impacts of significant weight loss during the developmental period, the negative shifts in behavior that parents could witness from the premorbid period to the adolescents being engaged in weight loss, and other indicators of negative psychosocial functioning, including social isolation and mood issues.

Complicating the activation of parents were concerns about potential treatment interference from other health care professionals involved in the care of the adolescent. These health care professionals mainly referred to the patient’s family physician or pediatrician, but also social workers, psychologists, dieticians, not directly involved with the child’s eating disorder treatment. Practitioners spoke to disciplinary differences within the medical field regarding the extent to which significant weight-loss was considered a marker of eating disorder pathology. Practitioners provided detailed accounts of pediatricians, family physicians, as well as other health care professionals (who were involved in an adolescent’s care, but were not members of the eating disorder team nor had expertise about eating disorders) providing counter-productive messages to adolescents and their families about the consequences of weight loss. Some practitioners further indicated that the broader health movement ‘blinded’ many health care professionals of the negative implications of extreme weight loss and that conflicting messages from these professionals could contribute to early treatment dropout among atypical AN adolescents and their parents. In addressing the anti-obesity movement and its impacts on atypical AN identification, one practitioner stated:

Yes, so the number one thing that I think is the issue is the highly publicized obesity epidemic…You know, pediatricians are terrified about this. They are coached to identify anyone tracking the 85^th^ percentile, regardless of their history. Parents are scared, schools are scared…Along with that there is a huge stigma. I think there are parents who – if their kid is overweight – that is the worst possible thing that could happen. I think that is getting worse because now it means you’re letting your kid be unhealthy and they are going to die… (Par 12, pg. 3)

This practitioner’s account emphasizes the complexities of atypical AN identification. Namely, that practitioners were concerned that generalists could have ‘blinders’ to atypical AN onset among adolescents who were premorbidly overweight or obese because the express goal of their work for the child has been focused on mitigating health risks associated with obesity.

#### Determining the goal weight range for adolescents with atypical AN

Although two of the 23 practitioners indicated that weight gain was not always required for atypical AN adolescents, *all* of the practitioners indicated that when it is required, identifying the goal weight range for these adolescents is the most salient challenge when delivering FBT. In particular, practitioners described that there were clear guidelines with respect to weight restoration when delivering FBT for typical AN adolescents (e.g., functional improvement in medical, growth, behavioural and cognitive domains), but not for their atypical peers. Practitioners grappled with the extent to which it was necessary to pursue weight restoration to the adolescents’ pre-morbid weight status:

I think the fact that some of what would be our typical calculations of things: calculating expected body weight, calculating roughly where a period might return, those kinds of things, can be more vague in the atypical cases. I feel like we either don’t know or understand exactly, whereas in an anorexia nervosa case, we don’t know obviously exactly when a period is going to return, but we have a pretty good sense generally of returning to a previous weight, maybe plus a few pounds. I feel like in these [atypical] cases, that can be less predictable, I feel like we have less of an understanding…we are really experimenting with what is the right weight? (Par 18, pg. 2)

Similarly, practitioners noted that in their experience of delivering FBT, full-weight restoration had been necessary for both adolescents with typical *and* atypical AN in order to appropriately address eating-disordered cognitions. Practitioners articulated debates within and across their teams about the necessity for full weight restoration when working with adolescents with atypical AN, and in some cases, practitioners identified this as a contentious issue that could complicate the activation of parents and adolescents to engage in treatment. Furthermore, the message of ‘full weight restoration’ was perceived by some practitioners as contributing to early treatment dropout in this patient population. For example, one practitioner stated:

There’s like some gray area and difference of opinion about the need for weight restoration or the degree of weight restoration required. Especially with some of the literature coming out indicating that maybe we don’t need to push kiddos that were formerly at the 85^th^ or 95^th^ percentile all the way back up to that same level. But yet, our medical team tends to still set their target weights around you know the 75^th^ or 85^th^ percentile, so there’s kind of conflicting opinions about that, but in my experience usually some degree of weight restoration is required. (Par 04, pg.1)

## Discussion

The present study explores practitioner perspectives on FBT and its applications to children and adolescents with atypical AN. The findings of our study indicate that there were a variety of factors influencing how practitioners classified atypical AN with diagnoses most often made *in relation* to cases that did not meet the defined weight criterion for typical AN. Practitioners in our study also expressed marked uncertainty around how to operationalize atypical AN, as there are currently no defined criteria in the DSM-5 around “significant weight loss” and “significant low weight” for the diagnosis for atypical AN. In response, some researchers have operationalized atypical AN in their studies using guidelines from the Society for Adolescent Health and Medicine [6, 29]. For example, Sawyer and colleagues [6] defined atypical AN in their adolescent participants according to those who lost more than 10% of their prior “healthy” body weight and were ≥ 90% of median body mass index (mBMI) for their age and gender. On the other hand, weight loss of even 5% can signify ED psychopathology [30]. However, for the practitioners in our study, the weight criterion constituted only one aspect of the classification of atypical AN, and practitioners often considered medical and psychological markers in addition to weight for diagnosis but lacked clarity about how to accurately diagnose atypical AN. The practitioners in our study were uncertain about the weight criterion that should be used – if at all – to differentiate atypical AN from typical AN. Setting aside the practice differences that depend on the patient presenting with atypical or typical AN, nearly all practitioners reflected that the length and phases of FBT were adapted depending on a number of factors; this represents a similarity between the application of FBT for both adolescents with atypical and typical AN. Practitioners adapted the duration of FBT phases depending on whether adolescents with atypical AN presented with a higher premorbid weight status, or if they required more time with families to establish acceptance of the treatment goals (such as weight gain and normalized eating).

When working with adolescents with atypical AN, practitioners described how they adopted *and* adapted manualized FBT for adolescents with atypical AN. Practitioners maintained fidelity to FBT in relation to externalization of the illness, completion of the family meal, but reported conflicting opinions about achieving weight restoration for young people with atypical AN. For the practitioners in our study who reported weight restoration as a necessary component of treatment, it might be possible that these practitioners are practicing this way because they always do (i.e., as part of their treatment modality); alternatively, another possibility is that their clinical judgement and experiential expertise is indicating that weight gain must happen for adolescents with atypical AN. Furthermore, practitioners detailed important nuances in utilizing the weight chart during treatment. While some practitioners in our study emphasized the importance of weight restoration as an indicator for recovery, others focused on individual weight trajectories and adjusted the scale of weight graphs to divert adolescents’ attention away from any discordance between the adolescent’s weight and population norms. In doing so, practitioners consistently described their adaptations to the graphing process as one that was attuned to the sensitivities of atypical AN adolescents and their families. Hughes and colleagues [19] discuss the importance of weight gain versus stabilization in the cognitive recovery of adolescents with atypical AN, acknowledging that many health care providers often hesitate to implement full weight restoration given the health risks associated with being overweight/obese. Similar to what practitioners noted in our study, these authors question the use of weight cut-offs and normed weight curves in practice, instead recommending the use of individual weight trajectories for identifying adolescents with atypical AN who have deviated from their original growth curves [19]. It is important to note that only two practitioners in our study highlighted their concern about reinforcing the ‘normalcy’ of the adolescent’s current weight status while presenting the individual weight trajectories, especially if this decreased the sense of urgency for the adolescent and their family around the ED. However, this concern was not consistent among the practitioners in our study.

Evidently, there is disagreement within the field regarding weight restoration for those with atypical AN. Future qualitative studies should respond to the use of weight restoration for adolescents with atypical AN, as practitioners who typically implement FBT assume that weight gain is necessary and embed this into their FBT practice [27]. In addition, future research should address how much weight gain is necessary for adolescents with atypical AN. The literature clearly demonstrates that when one’s brain is starved, cognitive engagement and effectiveness of psychotherapy is of limited success [31, 32]. However, adolescents with atypical AN may be at a weight that allows them to engage in psychotherapy, negating the idea that putting on additional weight is warranted for this population. Adding to this complexity, the issue of re-feeding to increase cognitive engagement in treatment did not systematically emerge in our data with atypical AN patients.

Finally, practitioners in the current study described significant practice challenges related to adapting FBT for adolescents with atypical AN. First, practitioners described challenges in legitimizing the ED diagnosis during treatment. This finding echoes the literature on the treatment of atypical AN using manualized FBT. Specifically, Hughes and colleagues [19] state the challenges in engaging families of adolescents with atypical AN in treatment when parents interpret the physical manifestations of the disorder as less threatening or severe. In contrast, the treatment approach of typical AN generally relies on markers of malnourishment and medical instability in establishing family engagement [19].

In addition, there was concern among practitioners when they worked other health professionals who may not have perceived the weight loss or changes in eating as problematic or unhealthy. Current research on pediatric care for adolescents with EDs highlights the need for physicians to better understand the principles of FBT in order to deliver appropriate care for patients with AN and their families. Training in this area could allow physicians to initiate preliminary FBT for adolescents as they wait for further consultation from ED specialists [33]. Even at a basic level, a general increase in primary and other health care practitioner knowledge regarding the features and treatment of EDs may be instrumental in preventing counterproductive messaging regarding weight loss, and may help mitigate the eating-disordered behaviors of adolescents with atypical AN prior to needing specialized eating disorder care.

The results of this study must be considered relative to the limitations. Practitioners based their perceptions and experiences on their work with atypical AN adolescents in their practice, a group which often makes up a smaller proportion of their eating disorder patients. Despite representing a small subset of patients, practitioners participating in the current study reported seeing between two to 50 families and youth with atypical AN using a FBT framework. That said, the FBT practitioners recruited for our study practiced in a variety of settings, including tertiary care programs, community-based organizations, and private practice, yielded a diverse sample with extensive expertise in implementing FBT in youth. Further, it would be naïve to overlook that practitioners may have their own implementations of FBT with typical AN, making these variations in atypical AN perhaps less meaningful or systematically different. Thus, it is important for future research to differentiate variations in the application of FBT for typical versus atypical AN. Finally, as this study did not set out to determine the effectiveness of FBT for adolescents with atypical AN, findings cannot be used to directly inform practice changes. Instead, reflections from practitioners were illustrated, which can be indirectly utilized to further dialogue and innovative research design, while maximizing the incentive for studies and trials on effective treatment for adolescents with atypical AN.

## Conclusions: research and clinical implications

Considering research on FBT practitioners’ perspectives working with adolescents with atypical AN is nascent, there are several recommendations for future research and practice that are imperative to improving outcomes for adolescents with atypical AN. As mentioned previously, many practitioners spoke of their relative inexperience in adapting this model for atypical AN, and reported a lack of knowledge around how to reconcile particular treatment outcomes (i.e., the need for weight-gain, goal weight, weight restoration versus stabilization, resumption of exercise, et cetera) between typical and atypical AN. Although addressing issues of treatment adaptation represents an important future step, more clinical clarity is required in differentiating between adolescents with atypical versus typical AN while reconciling treatment objectives and underlying principles. Lack of clarity around the definition of ‘significant weight loss’ and weight recovery for atypical AN was a consistent challenge, highlighting the need to study and develop other methods to assess degree of malnutrition. Thus, there is a need for further research to investigate the feasibility, efficacy, and treatment outcomes of the FBT approach for adolescents with atypical AN, in order to develop *manualized* adaptations to the existing FBT model for atypical AN in the future. However, it is critical to note that in FBT for atypical AN and typical AN the psychological issues related to these disorders are similar including a strong drive for weight loss and persistent irregular and eating. Recovery is about more than just weight restoration and should encompass medical stabilization, a return to normal eating and improved health and well-being. A misplaced effort on weight restoration when not needed in an adolescent with atypical AN may unnecessarily lead to a longer phase 1 of FBT. Doing so may mitigate the variability in approaches to treatment for atypical AN and produce an effective standardized treatment analogous to FBT for AN. Moreover, the reflections of the FBT practitioners interviewed reflected a possible need for increased training to meet the needs of this population. Overall, it seems that more work is necessary in this area in order to provide targeted clinical practice recommendations.

In pursuit of providing rigorous treatment for atypical AN, FBT practitioners should work closely with other health care providers (e.g., allied health care professionals, primary care physicians, pediatricians, et cetera) to ensure consistent messaging and underscore the impacts of extreme weight loss and weight restoration *despite* the adolescent’s weight at presentation. Finally, highlighting the similarities in treatment outcomes and psychological comorbidities between typical and atypical AN, along with other psychosocial indicators of eating disorder psychopathology may be imperative to increase urgency around the need for FBT.

## Abbreviations

AN: Anorexia nervosa
DSM-5: Diagnostic and Statistical Manual for Mental Disorders – 5^th^ Edition
BMI: Body mass index
FBT: Family based treatment
mBMI: Median body mass index
PI: Principal investigator

## Footnotes

1 Note on reflexivity (i.e., critical appraisal of the way in which the researcher’s background may have influenced the conduct of the research). The PI of this study is a registered social worker with over 20 years of clinical experience in both tertiary care and community-based settings. She has a longstanding research interest in eating disorders and delivers family-based treatments, group therapy and individual counselling for adolescents and adults with mental health issues.
